# Drug-Drug Interactions at Organic Cation Transporter 1

**DOI:** 10.3389/fphar.2021.628705

**Published:** 2021-02-17

**Authors:** Shiwei Zhou, Sujuan Zeng, Yan Shu

**Affiliations:** ^1^ Key Laboratory of Oral Medicine, School and Hospital of Stomatology, Guangzhou Medical University, Guangzhou, China; ^2^ Department of Pharmaceutical Sciences, School of Pharmacy, University of Maryland at Baltimore, Baltimore, MD, United States; ^3^ Department of Thyroid Surgery, The Second Xiangya Hospital, Central South University, Hunan, China

**Keywords:** organic cation transporters, OCT1, substrate, inhibitor, drug-drug interaction

## Abstract

The interaction between drugs and various transporters is one of the decisive factors that affect the pharmacokinetics and pharmacodynamics of drugs. The organic cation transporter 1 (OCT1) is a member of the Solute Carrier 22A (SLC22A) family that plays a vital role in the membrane transport of organic cations including endogenous substances and xenobiotics. This article mainly discusses the drug-drug interactions (DDIs) mediated by OCT1 and their clinical significance.

## 1 Introduction

Drug-drug interactions (DDIs) are among the critical factors in determining clinical drug disposition and response. DDIs refer to the changes in toxicity, pharmacokinetics or pharmacodynamics of a drug when two or more drugs are applied simultaneously or sequentially (Palleria et al., 2013; Prueksaritanont et al., 2013; Koepsell, 2015). DDIs on one hand can enhance the efficacy of a drug and on the other hand may reduce the efficacy or even lead to toxic reactions to a drug (Palleria et al., 2013; Sun et al., 2016; Niu et al., 2019). Movement of endogenous and exogenous chemicals across the biological membrane is usually mediated by transporter proteins that play a central role in the physiological function, pharmacological action, and elimination fate of these compounds. Drug transporters usually have extensive binding affinity toward a broad spectrum of small molecule substrates and inhibitors, suggesting their important role in DDIs (Girardin, 2006; Koepsell, 2015; Liang et al., 2015). Nowadays, more and more attention has been paid to the DDIs mediated by drug transporters. Transporter-mediated DDIs affect pharmacokinetics and pharmacodynamics, especially drug absorption and elimination (Giacomini et al., 2010; Liu et al., 2015). Drug transporters exist in almost all organs of human body, mainly in the brain, intestinal tract, kidney, liver, and lung (Liu and Pan, 2019).

The human organic cation transporter 1 (hOCT1), encoded by the *SLC22A1* gene, is highly expressed in the liver and possesses a broad substrate specificity (Koepsell et al., 2003). Approximately 40% of prescription medicines are organic cations (Neuhoff et al., 2003; Koepsell, 2020). The disposition of more than 120 drugs has been related to the activity of OCTs including OCT1-3 (Nies and Schwab, 2010). OCT1 function is thus closely related to pharmacotherapy of various diseases including cancer, cardiovascular and cerebrovascular diseases, digestive system diseases, substance addiction and CNS diseases. Because OCT1 can also transport certain endogenous metabolites, its activity may also be of great significance to the maintenance of homeostasis in the body (Nies et al., 2011b; Lozano et al., 2013; Brosseau and Ramotar, 2019; Li et al., 2019). Herein the physiological and pharmacological effects of OCT1 are briefly introduced, followed by a focused review on DDIs mediated by OCT1.

## 2 Molecular Cloning and Characterization of Organic Cation Transporter 1

OCT1 is a member of the Solute Carrier (SLC) Family 22 responsible for the uptake of numerous organic cations, anions and zwitterions, across the plasma membrane (Koepsell, 2013). Rat OCT1 (rOCT1) was the first cloned member of the SLC22A family. rOCT1 was cloned in 1994, and hOCT1 in 1997 by Koepsell group (Grundemann et al., 1994; Gorboulev et al., 1997). The human *SLC22A1* gene encoding hOCT1 is located on chromosome 6q26 and consists of 11 exons and 10 introns (Koehler et al., 1997). The human OCT1 protein has 554 amino acids. Like most transporters in the SLC22A family, it is composed of 12 α-helical transmembrane domains (TMDs) with intracellular N- and C-termini (Shu et al., 2003; Koepsell, 2013; Lozano et al., 2013). There is a large glycosylated extracellular loop between the TMD 1 and TMD 2, which can form disulfide bonds for protein oligomerization. In addition, between the TMD 6 and TMD 7, there is an intracellular loop with consensus sites that can be phosphorylated by several protein kinases. These glycosylation and phosphorylation sites are associated with the regulation of transport functions by regulatory proteins such as protein kinases A&C (Ciarimboli and Schlatter, 2005; Shu, 2011; Brosseau and Ramotar, 2019).

## 3 Distribution and Function of Organic Cation Transporter 1 in Human Tissues

**FIGURE 1:**
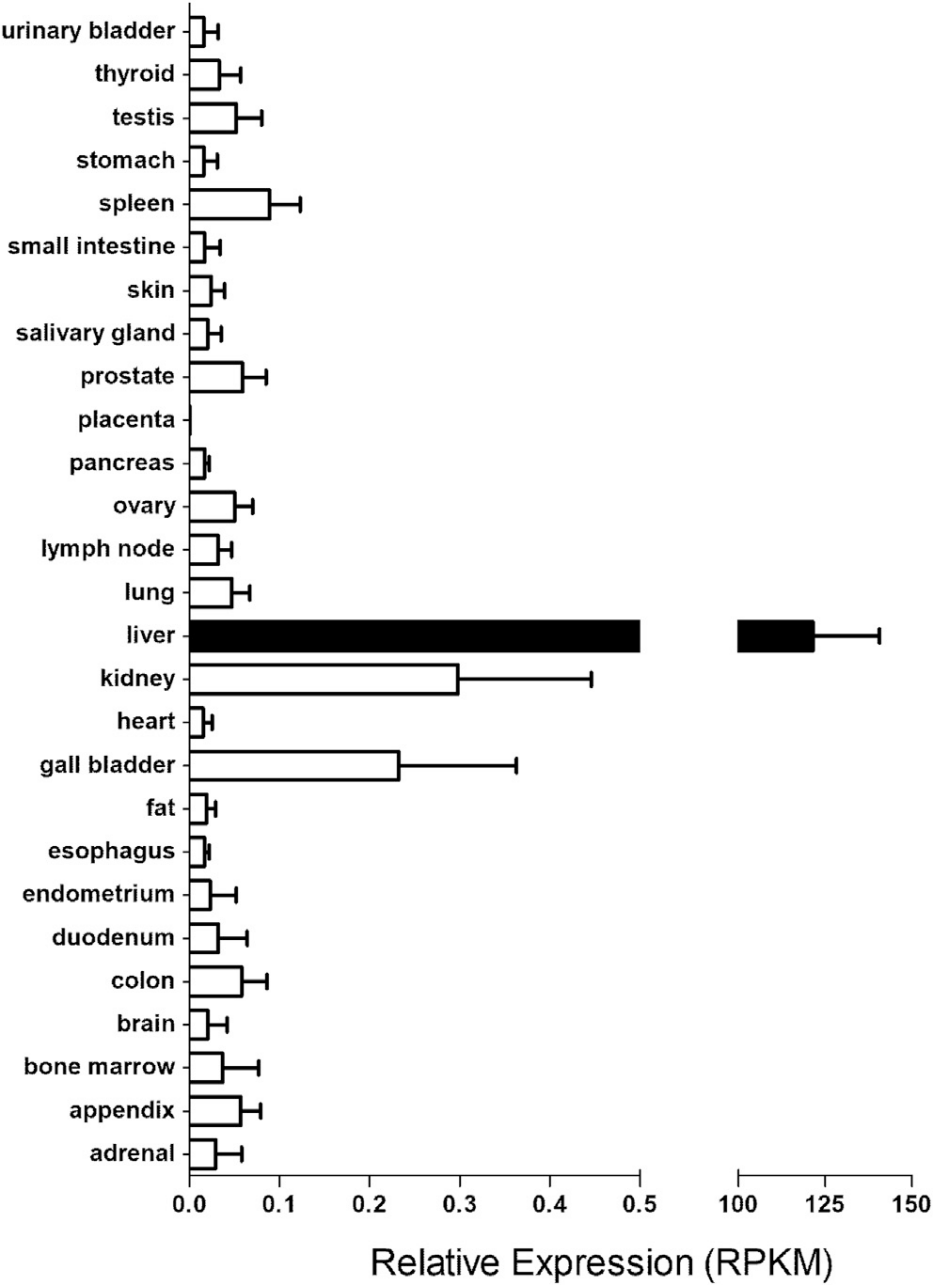
The transcript levels of *SLC22A1* gene in major human tissues. The RNA sequencing data for human tissues were retrieved from https://www.ncbi.nlm.nih.gov/gene/6580. RPKM stands for the Reads Per kilobase of transcript, per Million mapped reads in RNA sequencing, which is a normalized unit of transcript expression.

The importance of hOCT1 in drug disposition and response is implicated by its tissue expression pattern and cellular location. Although hOCT1 is widely distributed in human tissues, it is primarily expressed in the liver (Koepsell et al., 2003) (Figure 1). In hepatocytes, it has been located at the sinusoidal (basolateral) membrane. Of note, it is less expressed in cholangiocytes as compared to hepatocytes in the liver (Nies et al., 2009). In the intestine, there is evidence from immunolocalization and pharmacokinetics (PK) studies in support of hOCT1 expression in the basolateral membrane (Muller et al., 2005). However, this has been challenged by other results which supported that hOCT1 and mouse OCT1 (mOCT1) were actually expressed in the apical membrane of intestinal epithelia cells (Han et al., 2013). Further investigation is needed to settle this dispute. In the kidney, while rOCT1 has been reported to be located to the basolateral membrane of epithelial cells in the proximal tubules (Karbach et al., 2000; Sugawara-Yokoo et al., 2000), there is immunohistochemistry evidence supporting the location of hOCT1 in the apical membranes of proximal and distal tubules (Tzvetkov et al., 2009). In the lung, OCT1 is located in the lumen (apical) membrane of ciliated cells (Lips et al., 2005) and bronchial epithelial cells (Mukherjee et al., 2012). In addition, OCT1 has been reported to be expressed on the luminal side of brain microvessel endothelial cells (BMECs) (Lin et al., 2010), olfactory and nasal respiratory tissues (Chemuturi and Donovan, 2007), ovary, prostate, testis (Jung et al., 2008), cardiomyocytes (Rossato et al., 2011) and CD4^+^ cells of HIV-infected patients (Minuesa et al., 2008; Jung et al., 2013).

OCT1 is a poly-specific amphiphilic solute facilitator of transmembrane protein which bidirectionally mediates the transport of electrogenic organic cations across the plasma membrane in a manner independent of either Na^+^ or Ca^2+^ gradients (Busch et al., 1996; Gorboulev et al., 1997; Brosseau and Ramotar, 2019). OCT1 not only mediates the delivery of many cationic drugs and endogenous substrates into hepatocytes from the hepatic sinuses but also the release of organic cations from hepatocytes into the hepatic sinuses (Jonker and Schinkel, 2004; Koepsell et al., 2007; Nies et al., 2009). Consistent with its tissue expression patterns, OCT1 is also involved in the transport of certain substances in other organs. For example, it can regulate the secretion and absorption of organic cations in the small intestine (Koepsell, 1998), the reabsorption of ultrafiltration cations in the kidney (Koepsell et al., 1999), and the absorption of some drugs in the lung (Lips et al., 2005). Furthermore, OCT1 has been reported to promote organic cation crossing of the blood-brain barrier (BBB) (Lin et al., 2010), mediate the uptake of endogenous substrates into olfactory and respiratory mucosae (Chemuturi and Donovan, 2007) and the antiviral drugs into human immune cells (Minuesa et al., 2008; Jung et al., 2013).

## 4 Alteration of Organic Cation Transporter 1 by Liver Diseases

The liver predominantly expresses OCT1 and is the major organ responsible for drug metabolism in human body (Nishimura and Naito, 2005). A growing body of evidence suggests that the expression and function of OCT1 changes in liver diseases, which could affect drug disposition in the body, not only by increasing the possibility of DDIs but also by enhancing the complexity of drug treatment (Schaeffeler et al., 2011; Lautem et al., 2013; Li et al., 2019). Compared to that in normal rat liver tissues, rOCT1 mRNA expression was decreased in the presence of cholestasis (Cherrington et al., 2004). Interestingly, in the early stage of liver fibrosis associated with hepatitis C virus (HCV) infection, the hOCT1 mRNA expression was significantly increased (Ogasawara et al., 2010); however it decreases during the aggravation of fibrosis (Hanada et al., 2012). In addition, the alteration in human OCT1 expression in miscellaneous tumor cells, such as hepatocellular carcinoma (HCC) cells and cholangiocellular carcinoma (CGC), has also been reported. Compared with adjacent normal liver tissue, the expression of OCT1 was significantly down-regulated in primary liver cancers originating from epithelial cells such as HCC, CGC, and hepatoblastoma (Herraez et al., 2013; Lautem et al., 2013; Namisaki et al., 2014). In HCC and CGC, the reduced expression of OCT1 was associated with advanced tumor stages and poor patient survival (Heise et al., 2012; Lautem et al., 2013). The decreased expression appears to be caused by DNA methylation in the promoter of the *SLC22A1* gene (Schaeffeler et al., 2011).

## 5 Organic Cation Transporter 1 Substrates and Inhibitors

OCT1 works to regulate the cellular uptake of substrates. The substrates of OCT1 are usually organic cations with one or two positive charges, or weak bases with positive charges at physiological pH (Koepsell et al., 2003). Some uncharged compounds such as cimetidine can also be transported under alkaline conditions. The molecular weight of non-substrate inhibitors for OCT1 is in general larger than those of substrates. Sometimes, multiple inhibitor molecules can bind to the transporter protein simultaneously (Koepsell et al., 2003; Nies et al., 2011b; Shu, 2011; Koepsell, 2020). Most, if not all, of the substrates and inhibitors of OCT1 reported in the literature are summarized in Table 1.

**TABLE 1:** List of substrates and inhibitors of OCT1. The related information is cited from Drugbank https://www.drugbank.ca/categories/DBCAT004550, https://www.drugbank.ca/categories/DBCAT004549 and the references of this review.

Drug category	Substrates	Inhibitors
Alkaloids	Coptisine, jatrorrhizine, epiberberine and berberrubine, nitidine chloride, monocrotaline, retrorsine	Nuciferine, berberine, retrorsine, anisodine, monocrotaline
Alpha-2A adrenergic receptor agonists	Uanfacine	Guanfacine
Alpha-blockers	Prazosin	Prazosin, phenoxybenzamine
Anesthetics		Cocaine, lidocaine
Antiarrhythic drugs	Verapamil	Procainamide, verapamil, disopyramide, quinidine, dronedarone, propafenone
Antibiotics	Amoxicillin	Levofloxacin, trimethoprim, moxifloxacin
Anticancer drugs	Cytarabine, nintedanib, oxaliplatin, picoplatin	Rucaparib, dacomitinib, gilteritinib, palbociclib, nintedanib, irinotecan, erlotinib, nilotinib, dasatinib, mitoxantrone, paclitaxel, tamoxifen, amsacrine
Anticoagulant drugs	Nafamostat	
Anticonvulsant drugs	Lamotrigine	Lamotrigine
Antidepressant drugs	Fluoxetine	Desipramine, fluoxetine, imipramine, amitriptyline, trimipramine, citalopram, fluvoxamine, maprotiline, nomifensine, paroxetine, reboxetine, nefazodone, imipramine
Antifungal drugs		Ketoconazole, itraconazole, clotrimazole, isavuconazole, griseofulvin
Antihistamine agents	Chlorpheniramine maleate, diphenhydramine	Chlorpheniramine, dexchlorpheniramine maleate, diphenhydramine
Antihypertensive drugs	Amiloride	Reserpine, doxazosin, amiloride, diltiazem, clonidine
Antimalarial drugs	Quinine, proguanil	Quinine
Antimuscarinic drugs		Atropine
Antiparasitic drugs		Pyrimethamine
Antiparkinson drugs	Pramipexole, amantadine	Amantadine
Antiplatelet drugs		Clopidogrel
Antiprotozoal drugs	Pentamidine, furamidine	Pentamidine, furamidine, eflornithine
Antipsychotic drugs	Sulpiride, amisulpride, haloperidol	Quetiapine, chlorpromazine, clozapine, levomepromazine, remoxipride
Antituberculosis drugs	Ethambutol, isoniazid, prothionamide, *para*-aminosalicylic acid	Pyrazinamide
Antitussive drugs		Carbetapentane
Antiviral drugs	Ganciclovir, acyclovir, amantadine, lamivudine, peramivir	Ganciclovir, acyclovir, saquinavir, nelfinavir, indinavir, ritonavir, darunavir, efavirenz, nevirapine, daclatasvir
Beta-2 adrenergic agonist	Fenoterol, formoterol, salmeterol	Formoterol, salmeterol
Nonselective beta adrenergic receptor blocker	Nadolol	Carvedilol, bucindolol
Bronchodilators	Ipratropium, salbutamol	Ipratropium, metaproterenol, salbutamol
Diuretics		Spironolactone
Endogenous compounds	Histamine, dopamine, choline, epinephrine, norepinephrine, spermine, spermidine, serotonin, noradrenaline	Prostaglandin, choline, guanidine
Experimental compounds	Acetylcholine, choline salicylate, rhodamine, tropane alkaloids, cycloguanil, 4-(4-(dimethylamino)styryl)-N-methylpyridinium, synephrine	Nicotine, choline salicylate, tropane alkaloids, N1-methylnicotinamide, creatinine, corticosterone
Flavonoids	Quercetin	
Histamine H3 receptor antagonists		Pitolisant
Hormone drugs		Progesterone, estradiol acetate, estradiol benzoate, estradiol cypionate, estradiol dienanthate, estradiol valerate, osilodrostat
Hypoglycemic drugs	Metformin, phenformin, buformin	Phenformin, linagliptin, repaglinide, rosiglitazone, sitagliptin
H2 receptor antagonists	Cimetidine, ranitidine, famotidine	Cimetidine, ranitidine, famotidine
Immunosuppressants		Cyclosporine
Janus kinase inhibitors (JAK inhibitors)		Peficitinib
Muscarinic antagonists	Trospium chloride, oxybutynin	Oxybutynin
Neuromuscular blockers	Pancuronium, tubocurarine, rocuronium	Pancuronium, tubocurarine, rocuronium
Opioids	Methylnaltrexone, morphine, hydromorphone, norlevorphanol, norfentanyl, noroxycodone, meptazinol, 3-methoxymorphinan, oxymorphone, dextrorphan	Dextromethorphan, dextrorphan, levorphanol, levomethorphan, dextromethorphan, meptazinol, sufentanil, tapentadol, pethidine, norlevorphanol, tilidine, fentanyl, N-desmethyltramadol, morphine, nortilidine, tramadol
Selective serotonin receptor agonists	Sumatriptan	
Serotonin 5-HT3 receptor antagonists	Ondansetron, tropisetron	Ondansetron
Uricosuric drugs		Probenecid
Vitamins	Thiamine	
Serotonin (5-HT)1F receptor agonists		Lasmiditan

## 6 Interaction of Organic Cation Transporter 1 with Clinical Medication (Figure 2)

Many drugs are present as cations at physiological pH. As the most abundant organic cation transporter in the human liver, OCT1 mediates the transport of many organic cationic drugs across the hepatocyte membrane and may play an important role in regulation of metabolism of many drugs (Koepsell et al., 2007; Shu, 2011). Two or more therapeutic drugs that are OCT1 substrates may be administered simultaneously or subsequently in clinical applications. Because the expression level of OCT1 is relatively constant, competition between these substrates can happen for their cellular transport *via* OCT1. Likewise, some endogenous substrates can also compete for the uptake of xenobiotic drug substrates (Brosseau and Ramotar, 2019). In addition to the competitive inhibition of one substrate by another, many compounds such as lidocaine, prazosin, cocaine and dasatinib, which are not substrates of OCT1, can inhibit the uptake of OCT1 substrates (Brosseau and Ramotar, 2019). The inhibitors of OCT1, like those of other transporter proteins, are generally classified as competitive and non-competitive, depending on how the compounds interact with the binding site at the transporter protein and subsequently the dissociation manner (Belzer et al., 2013; Chen E. C. et al., 2017; Boxberger et al., 2018). However, both competitive and non-competitive inhibition may result in DDIs of clinical significance.

There is abundant evidence supporting a role of OCT1 in DDIs pre-clinically; however, only a few DDIs between OCT1 inhibitors/substrates have been reported in human subjects. Notably, even for these clinical DDIs, a contribution from other transporters or mechanism may not be excluded. For example, metformin is the victim drug in all of these clinical DDIs (Table 2). However, metformin is a substrate not only for OCT1 but also for OCT2, OCT3, plasma membrane monoamine transporter (PMAT), serotonin reuptake transporter (SERT), and others (Graham et al., 2011; Gong et al., 2012). In particular, OCT2 plays an important role in the renal elimination of metformin (Kimura et al., 2005). Although the role of OCT1 in metformin disposition and efficacy in human subjects is well supported by genetic evidence (Mofo Mato et al., 2018), the perpetrator drugs could have affected the activities of other metformin transporters, which might also contribute to the observed DDIs.

**FIGURE 2:**
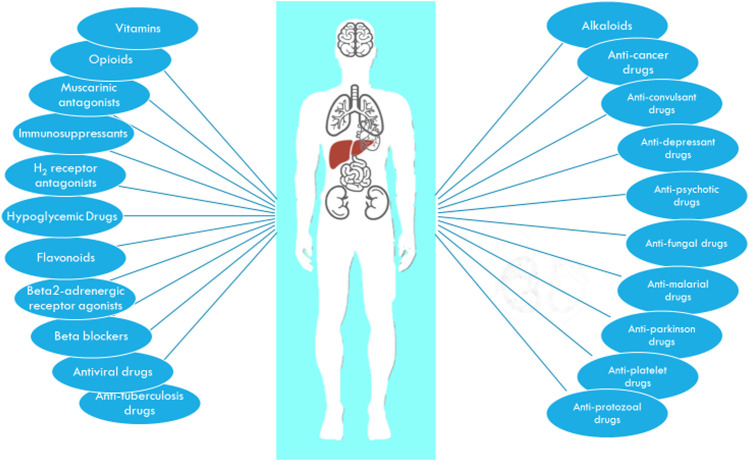
The major drug classes interacting with OCT1.

**TABLE 2:** Clinically relevant DDIs between OCT1 substrates/inhibitors with metformin. Note: Although OCT1 is assumed to play a role in these DDIs, the contribution from other transporters or mechanism cannot be ruled out.

Perpetrator drug	Victim drug	Effects
Isavuconazole	Metformin	The AUC and C_max_ of metformin were significantly higher with ivaconazole treatment Yamazaki et al. (2017)
Daclatasvir	Metformin	The number of adverse events increased in subjects that received both daclatasvir and metformin as compared to those receiving metformin alone Smolders et al. (2017)
Peficitinib	Metformin	The AUC, C_max_, and renal clearance (CL_R_) of metformin were reduced by peficitinib treatment, which is likely due to inhibition OCT1 and MATE1/2-K by peficitinib Shibata et al. (2020)
Opioids	Metformin	Opioids reduced the effect of metformin on the abundance of gut bifidobacterium, which is likely due to OCT1 inhibition by opioids Barengolts et al. (2018)
Verapamil	Metformin	Verapamil treatment reduced metformin’s ability to lower blood glucose, which is likely due to hepatic OCT1 inhibition by verapamil Cho et al. (2014)
Sitagliptin	Metformin	In subjects who did not reach the maximal goal of HbA1c with a sub-maximal dose of metformin, the addition of sitagliptin improved the glycemic response and glycated hemoglobin goals for metformin treatment Frias et al. (2019)
Rifampin	Metformin	Rifampin can up-regulate the expression of OCT1 in peripheral blood cells, increase the concentration of metformin in the blood and enhance the hypoglycemic effect of metformin Cho et al. (2011)
OCT1 inhibitors	Metformin	Concomitant use of medications, known to inhibit OCT1 activity, was associated with gastrointestinal side effects and intolerance of metformin Dujic et al. (2015)

The International Transporter Consortium, in collaboration with US FDA, has issued recommendations on transporter function assessment during drug development (Giacomini et al., 2010; Zamek-Gliszczynski et al., 2012; Brouwer et al., 2013; Hillgren et al., 2013; Tweedie et al., 2013). However, although the assessment of OCT1 is suggested by the European Medicines Agency (EMA), it has not been included in the industry guidance on DDI studies by FDA. According to the FDA guidance, an inhibitory *K*_*i*_ value of more than one-tenth of C_max_ has been suggested for the perpetrator drug to cause a clinically relevant interaction with another victim drug that is transported by the same transporter. It is expected that many drugs may significantly inhibit the activity of OCT1 at their clinical plasma concentrations. Specific DDIs mediated by OCT1 for major classes of drugs are reviewed below. Notably, the majority of these DDIs are either speculated from cellular findings or only evident at the preclinical level of animal studies. Therefore, additional clinical studies are highly needed to ascertain the role of OCT1 in various DDIs.

### 6.1 Alkaloids

Protoberberine alkaloids belong to isoquinoline alkaloids and are mainly found in the plants of *Fumariaceae*, *Berberidaceae* and *Papaveraceae* families, which include berberrubine, coptisine, jatrorrhizine, palmatine, epiberberine and corydaline. Proberberine alkaloids have been reported to possess a potent inhibitory effect on human OCT1/2/3 (Li et al., 2016). Moreover, coptisine, jatrorrhizine, epiberberine were found to be high-affinity substrates of OCTs, while berberrubine was a selective substrate for human OCT1 and OCT2, but not OCT3. The findings have provided useful information to understand the pharmacological effects of alkaloids or traditional herb medicines containing those alkaloids. In particular, the results suggest potential DDIs mediated by OCT1 between alkaloids and clinical used drugs.

Specifically, berberine, a quaternary ammonium alkaloid isolated from several plants, is the main effective component of rhizoma coptidis. Berberine has been reported to inhibit OCT1/2-mediated uptake of metformin in HEK293-OCT1 cells in a concentration dependent manner (Kwon et al., 2015). After intravenous administration of metformin with berberine, the initial blood concentration and Area Under Curve (AUC) of metformin were increased in rats, but the clearance rate and distribution volume of metformin were decreased. However, there was no change in the plasma concentration of berberine after administration with metformin. Shi *et al.* also reported a pharmacokinetic interaction between metformin and berberine (Shi et al., 2019). In their study, metformin and berberine were dosed by oral gavage. The plasma concentration and the AUC of metformin were decreased in rats that received berberine co-treatment compared to those that received metformin alone. Metformin was believed to be absorbed in the small intestine *via* OCT1 (Zhou et al., 2007; Graham et al., 2011; Han et al., 2015). rOCT1 inhibition by berberine may have contributed to the pharmacokinetic changes of oral metformin in rats.

Additional evidence suggests that the hepatic uptake of jatrorrhizine is mediated by a transport system belonging to the OATP and OCT families. In particular, prazosin, an OCT1 inhibitor, could potently inhibit OCT1-mediated uptake of jatrorrhizine in HEK-OCT1 cells. There are likely DDIs between the herbs containing jatrorrhizine and a substrate or inhibitor of OCT1 at the hepatobiliary disposition (Liang et al., 2020).

Nuciferine, one of the main active components of *Nelumbo nucifera Gaertn*, is considered as a promising agent for the treatment of obesity-related diseases. Li *et al.* characterized nuciferine as an inhibitor of OCT1 (Li et al., 2018). They found that nuciferine could reduce the concentration of metformin in the liver through mOct1 inhibition in mice. In addition, it could weaken the hypoglycemic effect of metformin. However, the effects of nuciferine on the hepatic concentration and hypoglycemic effect of metformin were present only for a period of time after nuciferine administration, suggesting that intermittent administration of nuciferine and metformin, if necessary, might prevent the DDI mediated by OCT1.

Nitidine chloride (NC) is a quaternary ammonium alkaloid with numerous pharmacological effects such as anticancer activity. However, NC also has hepatocellular toxicity. Li *et al.* reported that NC was a high affinity substrate of human OCT3 and OCT1 (Li et al., 2014b). The two transporters were believed to mediate the uptake of NC into hepatocytes and subsequently cause hepatotoxicity. Quinidine, an OCT1 inhibitor, could significantly reduce the hepatic uptake of NC and NC-induced toxicity in cultured primary rat hepatocytes. This study suggests OCT inhibition as a strategy to prevent clinical hepatotoxicity associated with NC use.

Monocrotaline (MCT) is a pyrrolizidine alkaloid and it has pneumotoxic and hepatotoxic effects in animals (Copple et al., 2002). Tu *et al.* demonstrated that MCT is a substrate and inhibitor of OCT1 and has a high affinity to the transporter (Tu et al., 2013). In MDCK-hOCT1 cells, OCT1 was found to play a vital role in the uptake and toxicity of MCT, and the inhibitor of OCT1, quinidine, could significantly inhibit the uptake of MCT, thereby reduce the MCT-induced toxicity.

### 6.2 Anti-Cancer Drugs

Oxaliplatin is an anti-cancer chemotherapeutic drug. Oxaliplatin has been characterized as an excellent substrate of human OCT1 and OCT2. The results by Zhang *et al.* have indicated that the cytotoxicity of oxaliplatin and its cellular accumulation could be inhibited by the OCT1 inhibitor disopyramide in MDCK-hOCT1 cells (Zhang et al., 2006). Furthermore, Buss *et al.* reported that pre- and co-incubation with atropine, an inhibitor of OCT1, significantly reduced oxaliplatin accumulation in drug-sensitive cells but not in drug-resistant cells (Buss et al., 2018). One possible mechanism is the alteration of transporter localization in the drug-resistant cells. The data suggest an association between OCT1 expression and oxaliplatin resistance.

Picoplatin is a third-generation platinum drug. It is very effective in the treatment of drug-resistant or refractory lung cancer. The chemical structures of picoplatin and oxaliplatin are similar. More *et al.* reported that the monoaqua complex of picoplatin (but not the diaqua complex) was a substrate of OCT1 (More et al., 2010). As similar for oxaliplatin in MDCK-hOCT1 cells, disopyramide reduced the cytotoxicity of picoplatin in lung cancer cell lines and the accumulation of platinum in HEK-hOCT1 cells. These studies have provided a foundation to delineate the role of OCT transporters in platinum-based chemotherapy and the related toxicity.

Mitoxantrone is an anthraquinone drug which is used to treat prostate cancer and leukemia. It has been demonstrated as an inhibitor of OCT1 (Gupta et al., 2012). In addition, Li *et al.* have shown that mitoxantrone could reduce the apical (AP) to basolateral flux of peramivir in Caco-2 cells (Chen J. et al., 2017). The reason might be that mitoxantrone could inhibit the activity of OCT1 which is expressed in the AP membrane of Caco-2 cells and plays a role in the influx of solutes in enterocytes. Thus, MCT administration may lead to a reduction in peramivir absorption.

Sorafenib, a multi-tyrosine kinase inhibitor (TKI), is considered as an effective targeting therapy for advanced liver cancer (Keating, 2017). OCT1 plays a role in the uptake of sorafenib into cells. The results by Al-Abdulla *et al.* indicated that sorafenib uptake was enhanced in the cells expressing hOCT1, which could be inhibited by the OCT inhibitor quinine (Al-Abdulla et al., 2019). Co-exposure with quinine suppressed not only hOCT1-mediated uptake of sorafenib but also sorafenib-induced cytotoxicity. In HCC patients treated with sorafenib, the protein expression of OCT1 at the plasma membrane was significantly associated with a beneficial response to sorafenib treatment. Interestingly, using the total healthy liver mRNA, there was no such association found (Geier et al., 2017). However, the relevance of OCT1 expression to sorafenib response remains controversial. Chen *et al.* recently reported that sorafenib was not a substrate of OCT1, and that the transporter was unlikely to participate in sorafenib disposition and influence its therapeutic effects in HCC (Chen et al., 2020).

Pazopanib is also a tyrosine kinase inhibitor. OCT1 has been reported to be responsible for the uptake of pazopanib in hepatocytes (Ellawatty et al., 2018). In addition, pazopanib is a potential inhibitor of OCT1 at clinically relevant concentrations. The unbound plasma concentration of pazopanib is slightly higher than the IC_50_ value of the pazopanib inhibiting OCT1-mediated uptake of metformin, suggesting a clinically relevant interaction between pazopanib and other drugs mediated by OCT1.

There are additional TKIs that have been reported as OCT1 inhibitors. Minematsu *et al.* demonstrated that erlotinib and nilotinib were potent inhibitors of OCT1 at clinically relevant concentrations (Minematsu and Giacomini, 2011). The two drugs could significantly inhibit metformin uptake mediated by OCT1 in HEK-hOCT1 cells. At a concentration similar to the clinically achievable unbound plasma concentrations, the two TKIs could inhibit the uptake of oxaliplatin. These data implicate clinical DDIs between TKIs and other OCT1 substrates or inhibitors.

OCT1 also interacts with additional anti-cancer drugs. For example, rucaparib is a potent small-molecule inhibitor of poly(ADP-ribose) polymerase enzymes that are important in cancer development and metastasis (Colombo et al., 2018). The results by Liao et al. indicated that rucaparib could potently inhibit human OCT1/2-mediated metformin uptake in cells (Liao et al., 2020). Because inhibition of OCT1/2 could decrease the uptake of metformin in the liver and its elimination in the kidney, and might reduce its hepatic anti-hyperglycemic action, there is a possible undesirable interaction between rucaparib and metformin in diabetic cancer patients who are treated by these two drugs.

### 6.3 Anti-Convulsant Drugs

Lamotrigine, an anti-epileptic medication, is also used to delay mood episodes in adults with bipolar disorder. Dickens *et al.* have reported that lamotrigine is a substrate and inhibitor of OCT1 and its transport into human brain endothelial cells can be mediated via OCT1 (Dickens et al., 2012). In addition, the anti-psychotic quetiapine, an inhibitor of OCT1, could inhibit the uptake of lamotrigine in the hOCT1-transfected cells. Importantly, the *in vitro* IC_50_ value for the inhibition was slightly lower than the steady state C_max_ in patients treated with quetiapine. Therefore, the concentration required to inhibit OCT1 in the patient is achievable after treatment with quetiapine. Although the effect of lamotrigine on cellular transport of quetiapine is uncertain, the potential DDI in patients between the two drugs should be considered.

### 6.4 Anti-Depressant and Anti-Psychotic Drugs

The BBB is an important physiological barrier between the central nervous system and the blood circulation. The antidepressants and antipsychotics must cross the BBB into the central nervous system to function. OCT transporters have been reported to be expressed in the BBB and could mediate the uptake of these drug (Amphoux et al., 2006) (Lin et al., 2010). Dos Santos Pereira *et al.* and Takano *et al.* reported that amisulpride and sulpiride were substrates of OCT1 (Dos Santos Pereira et al., 2014; Takano et al., 2017). Sekhar et al. also reported that amisulpride and haloperidol were transported by OCT1 (Sekhar et al., 2019). In addition, Kang *et al.* demonstrated that the neurotoxic pyridinium metabolites of haloperidol were substrates of OCT1, and pretreatment with OCT1 inhibitors verapamil, cimetidine, phenoxybenzylamine, and corticosterone could significantly inhibit the accumulations of these metabolites in Caco-2 cells (Kang et al., 2006). However, because certain drugs such as amisulpride can be a substrate of multiple transporters in different cells, sometimes OCT1-mediated DDIs involved these drugs may not be evident. For example, an inhibitor of OCT1, did not change the uptake rate of amisulpride in hCMEC/D3 cells but could inhibit the uptake of sulpiride, leading to a reduction of intracellular sulpiride accumulation (Dos Santos Pereira et al., 2014). Amantadine could increase the accumulation of amisulpride in bEnd.3 cells, but it had no effect in hCMEC/D3 cells. In contrast, prazosin could reduce the uptake of amisulpride in hCMEC/D3 cells but not in bEnd.3 cells. The accumulation of amisulpride was not affected by haloperidol in either cell line (Sekhar et al., 2017). Conversely, the uptake of haloperidol could be significantly reduced by amantadine, prazosin and amisulpride in Caco-2 cells (Kang et al., 2006).

Some other antidepressants and antipsychotics may have a potential inhibitory effect on OCT1 activity (Ahlin et al., 2008). Haenisch *et al.* reported that at the concentrations relevant to their clinical plasma levels, a wide range of pharmacologically different antidepressants and antipsychotics could inhibit the activity of human OCT1 by more than 20%, thereby likely interfering with the pharmacokinetics of OCT1 substrates in the liver, kidney and brain (Haenisch et al., 2012).

### 6.5 Anti-Fungal Drugs

Ketoconazole and itraconazole are antifungal medications, and they are generally regarded as clinically importantly CYP3A4/5 inhibitors (Varhe et al., 1994; Greenblatt, 2016). Recently, Vermeer *et al.* reported that ketoconazole and itraconazole are inhibitors of OCT1. The two drugs could inhibit the uptake of quinidine *in vitro* (Vermeer et al., 2016). However, they are not OCT1 substrates as the data indicated that they were not transported into the liver by hepatic OCT1 (Higgins et al., 2014).

Isavuconazole, a novel triazole antifungal prodrug, is used to treat invasive mucormycosis and aspergillosis (Maertens et al., 2016; Marty et al., 2016). In a clinical study, Yamazaki *et al.* has provided data in support of isavuconazole as an inhibitor of OCT1 (Yamazaki et al., 2017). Isavuconazole treatment could significantly alter the pharmacokinetics of metformin, such as the increase of its AUC and C_max_. Of note, isavuconazole PK was unaffected by metformin treatment.

### 6.6 Anti-Malarial Drugs

It has been reported that anti-malarials such as amodiaquine, primaquine, proguanil, pyrimethamine can significantly reduce the cellular activity of OCT1 (van der Velden et al., 2017). Moreover, proguanil and cycloguanil are found to be the substrates of OCT1 and other organic cation transporters including OCT2, MATE1 and MATE2-K. Because the endemic of malaria, HIV/AIDS and tuberculosis is always overlapped geographically, the incidence of co-infection among patients is high. Multiple drugs are required for the treatment of co-infection. As described above, the interaction with OCT1 is also common with anti-viral and anti-tuberculosis drugs. OCT1-mediated DDIs are expected in concurrent therapy for the co-infection.

### 6.7 Anti-Parkinson Drugs

Pramipexole is a dopamine receptor agonist, which is used to treat the symptoms of Parkinson disease. The drug has been reported as a substrate for rat OCT1 (Ishiguro et al., 2005). However, a study by Diao *et al.* indicated that pramipexole was not a substrate for human OCT1 (Diao et al., 2010). Instead, pramipexole was identified as a substrate of human OCT2 and OCT3. It is likely that the absorption of pramipexole in human intestine may be mediated by OCT3 and possibly OCT2. In addition, OCT2 and OCT3 may function to transport pramipexole in renal elimination and brain distribution, respectively.

### 6.8 Anti-Platelet Drugs

Clopidogrel (CP) is a widely used anti-platelet drug. It is either metabolized by cytochrome P450s into active metabolites in the liver or hydrolyzed by esterase to clopidogrel carboxylate (CPC). A study by Li *et al.* indicated that CP could strongly inhibit the uptake of lamivudine and amantadine mediated by human OCT1 in MDCK-hOCT1 cells (Li et al., 2014a). CPC could also significantly reduce the uptake of lamivudine in these cells but only had slight inhibition on the uptake of amantadine. The likelihood of clinical DDIs between CP and amantadine is expected to be low. On the other hand, although CP itself inhibits the uptake of OCT1 substrates such as metformin, lamivudine and amantadine, in consideration of the short duration of CP in the liver and a low plasma concentration, Li *et al.* thought that the DDI mediated by OCT1 between CP and those substrate drugs may not be serious *in vivo*. Future clinical observation is needed to confirm this postulation.

### 6.9 Anti-Protozoal Drugs

Pentamidine and furamidine are used to prevent severe lung infection in AIDS patients. They belong to a class of drugs called antiprotozoals. Ming *et al.* have shown that pentamidine and furamidine are good substrates of hOCT1. Ranitidine, a known OCT1 inhibitor, could significantly reduce the cytotoxicity of pentamidine and furamidine in CHO-hOCT1 cells (Ming et al., 2009). In addition, Sekhar *et al.* reported that pentamidine was a substrate for OCT1 transporter at the BBB (Sekhar et al., 2017). The OCT1 inhibitor amantadine could decrease the accumulation of pentamidine in hCMEC/D3 and bEnd.3 cell lines. However, another OCT1 inhibitor prazosin decreased pentamidine accumulation only in hCMEC/D3 cells, but not in bEnd.3 cells. Those OCT1 inhibitors may be non-specific, and other transporters might contribute to the cellular uptake of pentamidine as well. The significance of OCT1 in mediating a DDI between antiprotozoals and other drugs has yet to be confirmed.

### 6.10 Anti-Tuberculosis Drugs

The anti-tuberculosis (anti-TB) drugs are divided into two categories according to use frequency and efficacy: first-line and second-line anti-TB drugs. Among the approved drugs, the first-line essential agents that form the core of treatment regimens are rifampin (RIF), isoniazid (INH), and ethambutol (EMB) (Sotgiu et al., 2015).

Te Brake *et al.* reported that EMB is a substrate of OCT1. Moxifloxacin, which was characterized as a potent inhibitor of OCT1, could significantly inhibit the cellular transport of EMB (Te Brake et al., 2016). Later, Parvez *et al.* confirmed that EMB, amoxicillin, INH and prothionamide were novel substrates of OCT1 and as expected, the OCT1 inhibitor verapamil could strongly reduce their cellular uptake (Parvez et al., 2018). In addition, they found that the DDI indices of OCT1-mediated uptake of EMB and prothionamide were similar to that of verapamil, suggesting a strong *in vivo* potential of DDIs for these drugs with others.

Moreover, the DDI analysis by Pan *et al.* indicated that EMB has a strong potential for DDIs mediated by human OCT1 and OCT3 which are expressed in intestinal epithelial cells and hepatocytes. These DDIs may result in an altered absorption, distribution and excretion of the cationic drugs which are co-administered with EMB (Pan et al., 2013). For example, TB patients with coexisting diabetes or HIV might develop significant DDIs when co-treated with EMB and an OCT1/OCT3 substrate (e.g., lamivudine or metformin).

In addition, Parvez *et al.* reported that pyrazinamide, levofloxacin, and RIF could significantly inhibit OCT1-mediated metformin uptake in HEK-OCT1 cells (Parvez et al., 2016). With a static model-based approach to assess the correlation between the inhibitory potential of anti-TB drugs and the prognosis, they predicted a strong possibility of DDIs for these drugs interacting with other OCT1 substrate drugs *in vivo* on affecting anti-TB efficacy.

*Para*-aminosalicylic acid (PAS) is a second-line anti-TB drug used to treat multidrug resistant tuberculosis. The results by Parvez *et al.* indicated that PAS is a substrate of several transporters including OCT1 (Parvez et al., 2017). While they demonstrated that metformin effectively inhibited PAS uptake *via* OCT1, their estimated DDI index did not support the existence of clinical DDIs. They also found that omeprazole, lansoprazole, cimetidine, verapamil and quinidine could decrease the levels of OCT1-mediated PAS uptake *in vitro.* In contrast to that between PAS and metformin, the estimated DDI index values for the interaction between PAS and these OCT1 inhibitors were greatly higher than the cutoff and suggested possible clinical DDIs. The data are useful for future studies in patients to understand PAS disposition and clinical efficacy.

### 6.11 Antiviral Drugs

Many antiviral drugs have shown a binding affinity to OCT1 as substrates or inhibitors. Lamivudine, which is used to treat hepatitis B and HIV infection, belongs to a class of medications called nucleoside reverse transcriptase inhibitors (NRTIs). Human OCTs are characterized as important determinants of intracellular and plasma concentrations of lamivudine because they transport lamivudine and express in not only the organs of lamivudine disposition, such as liver and kidney, but also immune cells and excretory tissues that are critical to lamivudine action (Minuesa et al., 2009). Zalcitabine, another NRTI, has been demonstrated as a highly efficient substrate of OCT1 and OCT2 as well (Jung et al., 2008). Interestingly, the NRTIs abacavir and azidothymidine (zidovudine), the protease inhibitors nelfinavir, ritonavir, saquinavir, indinavir, and the anti-infective drugs pentamidine, trimethoprim are all high affinity inhibitors of OCT1 and OCT2. The concomitant administration of lamivudine and these potent OCT inhibitors is common in the regimen of highly active antiretroviral therapy (HAART). The DDIs may be of great significance in clinical practice, particularly for the pharmacokinetics, of lamivudine (Zhang et al., 2000; Jung et al., 2008; Minuesa et al., 2009; Jung et al., 2013; Arimany-Nardi et al., 2016). Consistently, Jung *et al.* documented that the addition of OCT1 and OCT2 inhibitors such as ritonavir and nelfinavir could reduce the accumulation of lamivudine in the CD4 cells of HIV-infected patients (Jung et al., 2013).

Efavirenz is antiviral drug in another class of medications called non-nucleoside reverse transcriptase inhibitors. It decreases the amount of HIV in the blood. Efavirenz has been demonstrated as an inhibitor of OCT1 by using hOCT1-overexpressing MDCK and KCL2 cells. The drug could inhibit the cellular transport and intracellular accumulation of lamivudine (Moss et al., 2015; Ceckova et al., 2018). The possible DDIs should be considered when co-administering efavirenz to HIV patients with other drugs.

Daclatasvir is used in combination with other medications to treat hepatitis C infection. Daclatasvir is a reversible and time-dependent inhibitor of OCT1 and OCT2 in cellular studies (Gandhi et al., 2018). However, Smolders *et al.* demonstrated that daclatasvir did not affect PK and PD parameters of the OCT1 substrate metformin in healthy subjects (Smolders et al., 2017). Interestingly, when daclatasvir was combined with metformin, the number of adverse events increased in human subjects. It has been suggested to monitor the adverse events during the treatment of type 2 diabetes mellitus (T2DM) patients with HCV infection under the combination treatment of daclatasvir and metformin.

In addition, Takeda *et al.* found that human OAT1 and hOCT1 are responsible for the renal transport of acyclovir and ganciclovir (Takeda et al., 2002). Caution should be taken when these antiviral drugs are used in conjunction with other drugs that share the same transporters for urinary tract excretion. Concomitant administration of these drugs may cause an increase in their plasma concentrations, leading to adverse drug reactions.

### 6.12 Beta-Adrenergic Receptor Blockers

Nadolol is a beta blocker that can be used alone or in combination with other drugs to treat high blood pressure. It is also used to prevent angina. Misaka *et al.* reported that nadolol was a substrate of human OCT1 and that OCT1-mediated nadolol uptake could be inhibited by cimetidine and trimethoprim *in vitro* (Misaka et al., 2016). In addition, carvedilol, another beta blocker, could inhibit metformin uptake mediated by human OCT1 and mouse OCT1 (Guo et al., 2018). These data will contribute to future human studies on OCT1-mediated DDIs involved beta blockers.

### 6.13 Beta2-Adrenergic Receptor Agonists

Beta-2-adrenergic agonists are first line agents in the treatment of asthma and other pulmonary disorders, such as chronic obstructive pulmonary disease. In a study by Salomon *et al.*, β2- adrenergic agonists such as salbutamol sulfate, formoterol fumarate, and salmeterol xinafoate were found to be substrates and inhibitors of OCT1 in human respiratory epithelial cells (Salomon et al., 2015). They demonstrated that the cellular uptake was mediated by hOCT1 in a time- and concentration-dependent manner for salbutamol, which was sensitive to inhibition by the OCT1 inhibitor verapamil. There was expression of hOCT1 and other organic cation transporters in human pulmonary epithelial cells. Therefore, OCT1 may be involved in the pulmonary disposition of beta2-adrenergic receptor agonists. Certain non-steroidal anti-inflammatory drugs (NSAIDs) were found to effectively inhibit the activity of OCT1 in leukemic cells (Wang et al., 2012). Mamlouk *et al.* found that the uptake of salbutamol was decreased in the presence of NSAIDs and proposed that NSAIDs could inhibit the absorption of salbutamol across the bronchial epithelium via the effects on OCT transporters (Mamlouk et al., 2013). In consideration of the highly polymorphic *SLC22A1* gene and a wide spectrum of substrates and inhibitors for this transporter protein, the DDIs mediated by OCT1 between drugs of this class and others may be clinically important.

### 6.14 Flavonoids

Flavonoids, such as phloretin and quercetin are secondary plant metabolites that can be found in different vegetables and fruits. Some flavonoids have been reported to possess health protective effects against cancer and cardiovascular diseases. There are studies indicating that quercetin is not a potent OCT1 inhibitor (Mandery et al., 2012; Glaeser et al., 2014). However, quercetin was characterized as a substrate of OCT1. In HEK293-hOCT1 cells, the uptake of quercetin could be significantly reduced by the OCT1 inhibitors such as amipamine, quinidine, and trimethoprim. There is also evidence in support of flavonoids as OCT1 inhibitors. Mimura *et al.* reported that hOCT1-mediated atenolol transport could be inhibited by rhestin and quercetin, which are the main components of apple juice, as well as several other flavonoids (Mimura et al., 2015). In a cellular study by Taur *et al.*, quercetin could inhibit the activity of the OCT system and reduce the intracellular accumulation of the substrate tetraethylammonium in LLC-PK1 cells (Taur and Rodriguez-Proteau, 2008). The flavonoids, such as quercetin, have the potential to alter the disposition profile of certain therapeutics by which cellular transport is mediated by cation transporters including OCT1.

### 6.15 Hypoglycemic Drugs

Diabetic patients frequently have to be treated with more than one drug. Among the anti-diabetic drugs, metformin is the most widely studied in relation to OCT1 function (Inzucchi et al., 2012). Previous reports mainly focus on the effect of metformin on the disposition of other drugs. However, recent studies have shown that some drugs used in combination with metformin in the clinical treatment of diabetes can also affect the disposal process of metformin through OCT1 (Dawed et al., 2019) (Table 2). In addition, studies have shown that the intestinal OCT1 and concomitant medications play a vital role in the gastrointestinal adverse effects of metformin (Dujic et al., 2016). When metformin is used in combination with proton pump inhibitors (PPIs), tricyclic antidepressants (TCAs) or codeine, the likelihood of metformin intolerance is greatly increased (Stage et al., 2016).

Naringenin is a colorless flavorless flavanone. Mata Mofo *et al.* reported that naringenin could up-regulate the expression of human OCT1, thereby improving the symptoms associated with diabetes (e.g., weight gain, heavy drinking, metabolic acidosis) (Mato Mofo et al., 2020). The diabetic patients treated with metformin may thus take grapefruit juice of which the predominant flavanone is naringenin. Stage *et al.* analyzed 32 drugs which may inhibit metformin transporters to assess the risk of early discontinuation of metformin (Stage et al., 2016). The odds ratio for early discontinuation of metformin was only found to be associated with codeine use. The results indicated that co-administration of codeine may be associated with a risk of early discontinuation of metformin.

Although deletion of *Slc22a1* gene in mice did not cause any apparent physiological defects, OCT1 can transport various endogenous metabolites, suggesting a physiological role by OCT1 activity in drug action (Chen et al., 2014). In addition to the transport of metformin, OCT1 may be a target for metformin (Chen et al., 2014; Chen et al., 2015). Metformin can competitively inhibit OCT1-mediated thiamine uptake in cells, resulting in reduced intestinal and systemic plasma thiamine levels, as well as liver thiamine levels. Modulation of thiamine levels *via* OCT1 by metformin might be critically important in its beneficial effects in treatment of diabetes, obesity, hepatic steatosis and cancer.

In recent years, gut microbiota has been linked to diabetes and other metabolic disorders. Metformin has an effect on the balance of gut microbiota. A study by Barengolts *et al.* found that the interaction between opioids and metformin had a significant effect on the abundance of bifidobacteria in the gut (Barengolts et al., 2018). Metformin treatment was associated with a decrease in the abundance of gut bifidobacterium in opioid users. In contrast, in the opioid non-users, metformin treatment was associated with an increase in the abundance of gut bifidobacteria. While the exact mechanism remains unclear, the authors hypothesized that opioids were inhibitors of OCT1, leading to a higher level of metformin in the blood and/or tissues which contributes to the observation. Response to metformin can be affected by other OCT1 inhibitors. Cho *et al.* indicated that verapamil could reduce metformin's ability to lower blood glucose, but did not affect its pharmacokinetics (Cho et al., 2014). One of the reasons is that verapamil likely act as a potent competitive OCT1 inhibitor, preventing metformin uptake into the liver. Interaction between verapamil and metformin in patients with hypertension and T2DM may thus affect their efficacy and safety. In addition, OCT1 inhibitors were regarded as important players in metformin gastrointestinal side effects experienced by up to 20–30% of patients (Dujic et al., 2015). The DDI between metformin and an OCT1 inhibitor could become even complex in individuals with *SLC22A1* genetic polymorphisms (Dujic et al., 2016).

Common genetic variation of the *SLC22A1* gene could reduce the transport of substrates such as metformin in the liver (Shu et al., 2007; Ahlin et al., 2011). Compared with fully functional hOCT1- reference (NM_003057), the polymorphic hOCT1 proteins such as M420del and R61C were more susceptible to the inhibition by inhibitors. Specifically, the uptake of metformin via hOCT1- M420del was subjected to more inhibition by clinically relevant concentrations of verapamil, as compared to the hOCT1- reference. The enhanced sensitivity to drug inhibition toward OCT1 variants may lead to an increased risk of DDIs in individuals with these variants.

There are additional reports on DDIs between metformin and clinical used drugs. Frias *et al.* reported that in subjects who did not reach the maximal goal of HbA1c with a sub-maximal dose of metformin, the addition of sitagliptin improved the glycemic response and glycated hemoglobin goals, while the safety and tolerability were similar with metformin treatment alone (Frias et al., 2019). A possible mechanism is that the inhibition of OCT1 by sitagliptin could reduce the phosphorylation of AMPK, the first step in metformin's action (Choi et al., 2010). In addition, Cho *et al.* found that rifampin can up-regulate the expression of *SLC22A1* gene in peripheral blood cells, increase the concentration of metformin in the blood and enhance the hypoglycemic effect of metformin (Cho et al., 2011). Rifampin could also increase renal tubule secretion of metformin. In patients with T2DM and tuberculosis, the interaction between metformin and rifampicin may thus affect drug safety and efficacy. On the other hand, because the most toxic side effect of metformin, lactic acidosis, is a dose-dependent effect, reducing the dose of metformin may reduce the risk of lactic acidosis.

*In vitro* evidence has also suggested that OCT1 may be able to mediate an interaction of metformin with other clinical drugs or diet supplements. For example, green tea and its most abundant catechin epigallocatechin gallate (EGCG) could inhibit the transport of metformin mediated by hOCT1 in cellular studies (Knop et al., 2015; Albassam and Markowitz, 2017). Interestingly, the inhibitory effect by green tea even exceeded that by EGCG. Bachmakov *et al.* also reported that the anti-diabetic repaglinide and rosiglitazone could significantly inhibit hOCT1-mediated metformin uptake in cells (Bachmakov et al., 2008).

Of note, DDIs *in vitro* may not necessarily translate into a clinical DDI. Recent *in vitro* studies have found that PPIs may interfere with the effectiveness of metformin (Nies et al., 2011a). However, Flory *et al.* has shown that the use of PPIs did not impair the effectiveness of metformin and that PPIs themselves had no significant clinical impact on glycemic control (Flory et al., 2015). Metformin was at least as effective in reducing glycosylated hemoglobin in patients with chronic PPIs treatment as in patients without PPIs treatment. Peficitinib, a pan-Janus kinase inhibitor, is used to treat rheumatoid arthritis (Takeuchi et al., 2016). Peficitinib has been shown to inhibit the uptake of metformin in hOCT1-overexpressing cells (Shibata et al., 2020). However, in clinical studies, the AUC, C_max_ and CL_R_ of metformin were only slightly reduced by peficitinib treatment in healthy male subjects. As metformin is a relatively safe and generally well tolerated by patients, the interaction between peficitinib and metformin may not be clinically important and metformin dose adjustment may be not required. However, further clinical studies in patients are always needed to confirm the assumption based on *in vitro* findings and those from healthy human subjects.

### 6.16 H_2_ Receptor Antagonists

Cimetidine, ranitidine and famotidine belong to a class of drugs called H_2_-receptor antagonists. These drugs have been reported as the substrates for OCTs but are used primarily as the inhibitors of OCTs in many studies (Barendt and Wright, 2002; Bourdet et al., 2005). Meyer *et al.* found that as a substrate or competitive inhibitor of OCT1, ranitidine could inhibit hOCT1-mediated uptake of morphine and metformin at clinically relevant concentrations (Meyer et al., 2017). In addition, the uptake of ranitidine was also affected by common genetic polymorphisms of *SLC22A1* gene. However, although co-medication of ranitidine significantly reduced the rate of renal clearance of trospium chloride, the oral absorption and distribution did not change in healthy subjects (Abebe et al., 2020). Because of potential effects by disease status and genetic polymorphisms on transporter function, the clinically relevant impact of ranitidine on the pharmacokinetics of trospium chloride and other drugs in patients remain to be further delineated.

### 6.17 Immunosuppressants

Cyclosporine A (CsA) is a large lipophilic cyclic polypeptide. It can prevent organ rejection after transplant and is used to treat rheumatoid arthritis and psoriasis. In a cellular study, CsA was identified as an inhibitor of hOCT1 (Panfen et al., 2019). In particular, the inhibitory potency of CsA against hOCT1-mediated metformin uptake was 50-fold higher with CsA pre-incubation as compared to co-incubation. Interestingly, the difference in inhibitory potency between pre-incubation and co-inhibition with CsA seemed to be substrate-dependent. The IC_50_ shift ranged from >1.2- to 50.2-fold with different substrates. While it would be interesting to understand the mechanism underlying the shift of hOCT1 inhibition by CsA with different incubation conditions, the potent and persistent inhibitory effect on hOCT1 after exposure to CsA implies hOCT1-mediated DDIs with other drugs in patients.

### 6.18 Muscarinic Antagonists

Trospium chloride (TC) is a muscarinic antagonist that is used to treat overactive bladder and symptoms of urinary frequency, urgency and incontinence (Wenge et al., 2011). TC is not completely absorbed from the gut. While it is widely distributed after absorption, it does not significantly pass the BBB (Bexten et al., 2015). TC can be eliminated from the kidney, liver, and intestine. It has been characterized as a substrate of several transporters including OCT1, P-glycoprotein, and OATP1A2. In cell studies, TC was taken up by human bladder urothelial cells through a mechanism that is susceptible to the inhibition by verapamil, an inhibitor toward several transporters. Although OCT1 may contribute to the disposition of TC, currently there is no evidence in support of any serious OCT1-mediated DDIs for this drug.

### 6.19 Opioids

Morphine, an opioid receptor agonist, has been determined as a substrate of OCT1 (Balyan et al., 2017). Zhu *et al.* has shown that both OCT1 and OCT2 can mediate the cellular uptake of morphine (Zhu et al., 2018). Moreover, irinotecan could alter the distribution of morphine *in vivo* in mice by inhibiting mouse OCT1 activity. In addition, cellular hOCT1-mediated uptake of morphine was found to be inhibited by a variety of inhibitors, including irinotecan, verapamil, ondansetron, imipramine, codeine, amitriptyline, tropisetron, fluoxetine, and clomipramine, at the concentrations relevant to those at the portal vein in patients receiving these inhibitors (Tzvetkov et al., 2013). Although the plasma concentrations of these drugs are too low to inhibit the activity of OCT1, these drugs may still have a potential to cause DDIs with morphine because their oral administration may result in a higher concentration in the hepatic portal vein. However, morphine and codeine by themselves may have very moderate inhibitory effects on OCT1-mediated drug uptake, due to their low portal vein concentrations following oral administration. Considering that those patients requiring morphine for pain relief commonly receive concomitant medications, clinicians should be aware that the therapeutic and/or toxic effects of morphine may be altered by the co-administrated inhibitors and/or substrates of OCT1, such as irinotecan.

### 6.20 Vitamins

Thiamine, also known as vitamin B1, is found in foods such as cereals, whole grains, beans, meat, nuts and peas. It plays an important role in the breakdown of carbohydrates from foods into intermediate metabolites needed by the body. Thiamine has been identified as a substrate of OCT1 (Kato et al., 2015). However, multiple transporters may mediate the hepatocellular uptake of thiamine. The hOCT1-mediated uptake of thiamine may be only physiologically relevant at high concentrations, whereas other transporters are responsible for thiamine uptake into the liver at typical blood concentrations (Jensen et al., 2020). In the intestine, while the absorption of thiamine has been reported to be mediated by thiamine transporters ThTr1 and/or ThTr2, there is also contribution by OCT transporters, most likely by OCT1 and/or OCT3 (Lemos et al., 2012). The findings of thiamine as an OCT1 inhibitor have implicated an interaction mediated by OCT1 between nutrients and drugs, especially in patients who have been chronically treated with certain drugs and under a special diet. For example, as discussed above, there is potential OCT1-mediated interaction between thiamine and metformin in T2DM patients (Chen et al., 2014).

## 7 Closing Remarks

In recent years, more and more attention has been paid to OCTs in the fields of clinical pharmacology and pharmaceutical research. Among these OCTs, OCT1 is widely distributed in different tissues with an extremely high level in the liver. A broad spectrum of substrates and inhibitors has been characterized for this transporter. Increasing evidence has indicated that OCT1 might be an important mediator for DDIs of clinical significance. However, the confirmed DDIs mediated by OCT1 in human subjects remain limited. A major reason is that an effective and convenient tool to probe OCT1 activity in humans has yet to be discovered and validated. OCT1 is highly polymorphic, with multiple common variants leading to functional alteration. The effort to study the DDIs of OCT1 substrates and inhibitors in the patients with different OCT1 genotypes may yield important clinical evidence in the near future. Current effort in characterizing the interaction of OCT1 with an increasing number of compounds will bring us valid probe drugs to assess OCT1 function in patients and lead to appreciation of its clinical importance in drug disposition and response. Our understanding of OCT1-mediated DDIs will eventually have an impact on optimization of pharmacotherapy in order to improve drug efficacy and avoid unnecessary DDIs.
